# Linking Methanogenesis in Low-Temperature Hydrothermal Vent Systems to Planetary Spectra: Methane Biosignatures on an Archean-Earth-like Exoplanet

**DOI:** 10.1089/ast.2022.0127

**Published:** 2023-03-31

**Authors:** Rhys Seeburger, Peter M. Higgins, Niall P. Whiteford, Charles S. Cockell

**Affiliations:** ^1^ UK Centre for Astrobiology, School of Physics and Astronomy, University of Edinburgh, Edinburgh, UK.; ^2^ Institute for Astronomy, University of Edinburgh, Royal Observatory, Blackford Hill, Edinburgh, UK.; ^3^ Max Planck Institute for Astronomy, Heidelberg, Germany.; ^4^ Department of Earth Sciences, University of Toronto, Toronto, Canada.; ^5^ Centre for Exoplanet Science, University of Edinburgh, Edinburgh, UK.; ^6^ American Museum of Natural History, New York, New York, USA.

**Keywords:** Biosignatures, Methods: numerical, Exoplanets and satellites: atmospheres

## Abstract

In this work, the viability of the detection of methane produced by microbial activity in low-temperature hydrothermal vents on an Archean-Earth-like exoplanet in the habitable zone is explored via a simplified bottom-up approach using a toy model. By simulating methanogens at hydrothermal vent sites in the deep ocean, biological methane production for a range of substrate inflow rates was determined and compared to literature values. These production rates were then used, along with a range of ocean floor vent coverage fractions, to determine likely methane concentrations in the simplified atmosphere. At maximum production rates, a vent coverage of 4–15 × 10^−4^ % (roughly 2000–6500 times that of modern Earth) is required to achieve 0.25% atmospheric methane. At minimum production rates, 100% vent coverage is not enough to produce 0.25% atmospheric methane. NASA's Planetary Spectrum Generator was then used to assess the detectability of methane features at various atmospheric concentrations. Even with future space-based observatory concepts (such as LUVOIR and HabEx), our results show the importance of both mirror size and distance to the observed planet. Planets with a substantial biomass of methanogens in hydrothermal vents can still lack a detectable, convincingly biological methane signature if they are beyond the scope of the chosen instrument. This work shows the value of coupling microbial ecological modeling with exoplanet science to better understand the constraints on biosignature gas production and its detectability.

## 1. Introduction and Background

**FIG. 1.**
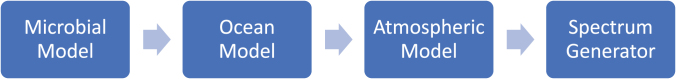
A basic outline of the process described in this work. NutMEG is used to generate a microbial model, driving the simulation of a bioreactor in the deep ocean. Outflows of gas from this are then used to compute atmospheric gas concentrations. These then feed into the spectral generator, which allows us to assess the performance of various instruments.

A growing and crucial area of astrobiology is the study of how microbial life influences exoplanetary environments and how we could use these processes to infer the presence of life beyond our solar system. This research is motivated by the increasingly regular discovery of rocky habitable zone exoplanets (Gillon *et al.,* 2017) and the promise of extensive spectroscopic observations of their atmospheres in the coming decades. Some recent studies have even suggested that terrestrial exoplanets in the habitable zone may exist in abundance, possibly around 40–60% of Sun-like stars (Bryson *et al.* , 2020)—though this may be an optimistic estimate due to assumptions made in extrapolating from Kepler data, as Neil and Rogers (2020) estimate this to be as low as ∼0.5%. This potential sample of exoplanets will offer an extensive test bed in the search for life but will require refined and thorough explanations of the interactions biological surface and subsurface activity can have with its observable atmosphere. In recent decadal surveys for astronomy, planetary sciences, and astrobiology, the National Academies of Sciences, Engineering, and Medicine (NASEM) have independently highlighted important overlaps in the next steps for both the astronomy and astrobiology communities, including for example the need to constrain the observable characteristics of inhabited planets, determine whether subsurface biospheres could ever be detected remotely, and avoid pitfalls such as false-negative or false-positive detection of life (NASEM, 2021, 2022). Therefore, in this report, we emphasize the interaction between microbial life and its environment, exploring how one affects the other. We then investigate the viability of using these effects to infer the presence of life on exoplanets via their atmospheric signatures using future observatories. As such, this work presents a unique bottom-up approach combining microbial simulations with atmospheric modelling and spectral generation. The process is summarized in Fig. 1.

The use of computer modelling to explore microbiological processes is not new (*e.g.,* Ferrer *et al.,* 2008; Amend *et al.,* 2011). Similarly, the effect of different gas concentrations on a planet's atmospheric spectrum as well as the presence of biosignatures and their detectability have both been the topic of detailed study (*e.g.,* Kaltenegger *et al.,* 2007; Krissansen-Totton *et al.,* 2018a; Meadows *et al.,* 2018; Rugheimer and Kaltenegger, 2018). However, so far little attempt has been made to directly link microbial ecology knowledge and modelling to atmospheric composition and biosignature detection in exoplanet science despite being an important step in proposed frameworks to validate a biosignature detection (Green *et al.,* 2021; Meadows *et al.,* 2022). In this report, we investigate the production of methane by methanogens in hydrothermal vent fields using a model, and we then investigate the subsequent detection of this methane in an anoxic atmosphere. We use these data to test the detectability of biologically produced methane on terrestrial exoplanets. We note here that we use the term “biosignature” to mean a chemical signature in an atmosphere that is consistent with likely having a biological source. There is no guarantee that a biosignature as such necessitates life to be present on the planet in question.

Methanogens are archaea characterized by their production of methane as part of their metabolism. They do this via a number of chemical pathways, broadly gathered into three groups defined by the molecules used for this metabolism: carbon dioxide, acetate, or other small organic compounds. As their metabolism is anaerobic (does not require oxygen), they can live in a wide variety of environments, including the deep ocean, sewage, and animals' intestines. This is also made possible by the fact that growth temperatures for methanogens cover a wide range, including psychrophilic (cold-loving) to hyperthermophilic (heat-loving) organisms. (Kim and Whitman, 2014). However, methanogenesis does not have high energy yields—only 0.5 to 2 moles adenosine triphosphate (ATP) per mole of substrate (Buan, 2018).

Some suggest that methane in conjunction with carbon dioxide can be used as a biosignature. This is because continuous methane production orders of magnitude greater than that of only abiotic sources is necessary to maintain the coexistence of carbon dioxide and methane over geological timescales. This amount of methane production requires a biological source (Pidhorodetska *et al.,* 2020). Krissansen-Totton *et al.* (2018b) calculate that atmospheric methane concentrations >0.1% imply source fluxes of >7 Tmol/year, which they deem “likely biogenic,” while concentrations above 1% imply source fluxes in excess of 50 Tmol/year, which are “very likely biogenic” when coexisting with carbon dioxide. In Section 2, we explore these thresholds in a little more detail. It is important to note that merely detecting high atmospheric concentrations of methane alone is not sufficient, as it is specifically the coexistence of methane and carbon dioxide that necessitates a high methane flux, and is thus more likely to have a significant biological component (Krissansen-Totton *et al.,* 2018b). In any case, it is also important to note that other unknown abiogenic processes could also contribute to a high flux of methane; thus it is most useful as a biosignature when co-detected with other biosignature species (Krissansen-Totton *et al.,* 2018b).

Hydrothermal vents are located on an ocean floor, commonly found at or near tectonic plate boundaries, where heat from magma chambers within the planet's crust causes geothermally heated water to be circulated through and discharged from the upper crust (Martin *et al.,* 2008). At hydrothermal vents, serpentinization, a geochemical process, is responsible for a significant fraction of the available molecular hydrogen and methane (Schulte *et al.,* 2006). Seawater circulating through the oceanic crust provides water, and carbon dioxide dissolved as bicarbonate. At high temperatures (150–200°C) Fe(II) in the rocks oxidizes to Fe(III) in these conditions, while reducing water to molecular hydrogen and producing hydrocarbons. This provides a source of molecular hydrogen, which can act as an electron donor for many redox couples employed by life (Sleep *et al.,* 2004).

In this article, we explore the journey of biogenic methane on a hypothetical Archean-Earth-like exoplanet, from the metabolism of methanogens in a hydrothermal vent system through an ocean and into the atmosphere. Along the way we note several obstacles which require specialist attention to properly describe the transport of biosignatures such as this. These include, for example, ocean transit and absorption, atmospheric transit and methanotrophy, among others. We then estimate the amount of hydrothermal vent coverage the planet would need to produce a spectrum with noticeably biogenic methane levels.

The detectability of methane depends on the telescope and instrument used. A larger methane concentration in the atmosphere will lead to a more noticeable difference in the spectrum when compared to a “non-biogenic” baseline, but any instrument used for detection needs to both cover the relevant wavelength range (which includes methane absorption lines) and be able to achieve a high enough signal-to-noise ratio. In this work, we compare several next-generation instrumental concepts and assess their capabilities for detecting a methane biosignature. LUVOIR (Large Ultraviolet/Optical/Infrared Surveyor) is proposed to launch in the late 2030s and consists of two concepts: LUVOIR A and LUVOIR B, with mirrors of 15 and 8 m diameter, respectively. It features the ECLIPS (Extreme Coronagraph for Living Planetary Systems), as well as HDI (High Definition Imager), LUMOS (LUVOIR Ultraviolet Multi-Object Spectrograph), and Pollux (a high-resolution UV spectro-polarimeter), which provide the technology necessary to perform high-precision astrometry and multi-object spectroscopy (Bolcar *et al.,* 2017; NASA, 2019). HabEx (Habitable Exoplanet Observatory) features a 4 m diameter telescope and is proposed to launch in the mid 2030s. Like LUVOIR, HabEx comprises several instruments: the HabEx Coronagraph (HCG), as well as a starshade (SS) with a diameter of 52 m for exoplanet imaging and characterization, the Workhorse Camera for multiple purposes including wide-field imaging and spectroscopy, and a UV spectrograph (Gaudi *et al.*, 2020).

Additionally, we attempt to predict the approximate performance of the LUVOIR/HabEx hybrid (LUVEX) suggested by the National Academy of Sciences' decadal survey (NASEM, 2021).

## 2. Materials and Methods

Our focus will be placed specifically on carbon dioxide–reducing methanogens, using molecular hydrogen as the electron donor in the following reaction: CO_2_ + 4H_2_ → CH_4_ + 2H_2_O.

To explore the effects of methanogens in hydrothermal vent fields on the atmosphere, environmental parameters of these vent fields have to be determined, the behavior of methanogen communities in these environments modelled, their methane output determined and the eventual accumulation of this methane in the atmosphere calculated. Then, spectra can be generated and compared in order to determine biosignature detectability. This process was broken down into individual steps.

Computer modelling of biogenic fluxes was achieved using the NutMEG package (Higgins and Cockell, 2020), and atmospheric spectra were created using the NASA Planetary Spectrum Generator (PSG) (Villanueva *et al.,* 2018). As a starting point, an Archean-Earth-like terrestrial exoplanet was assumed to help gauge reference values for a wide variety of parameters (Catling and Zahnle, 2020).

Table 1 contains a summary of the instrument parameters used in this work. A concise summary of fixed parameters assumed can be found in Table 2, and varying parameter spaces to be explored in Table 3, both at the end of this section.

**Table 1.** Instrument Parameters for HabEx and LUVOIR, Adapted from Checlair *et al.* (2021)

Parameter	LUVOIR A	LUVOIR B	HabEx SS	HabEx no SS
Diameter	15 m	8 m	4 m	4 m
Wavelength range	UV: 0.2–0.515 μm	UV: 0.2–0.515 μm	UV: 0.2–0.45 μm	UV: N/A
	VIS: 0.515–1.0 μm	VIS: 0.515–1.0 μm	VIS: 0.45–0.975 μm	VIS: 0.45–0.975 μm
	NIR: 1.0–2.0 μm	NIR: 1.0–2.0 μm	NIR: 0.975–1.8 μm	NIR: 0.975–1.8 μm
Resolution	UV: 7	UV: 7	UV: 7	UV: N/A
	VIS: 140	VIS: 140	VIS: 140	VIS: 140
	NIR: 70	NIR: 70	NIR: 40	NIR: 40
Exozodi level	4.5	4.5	4.5	4.5
Contrast	1 × 10^−10^	1 × 10^−10^	1 × 10^−10^	2.5 × 10^−10^
IWA [λ/D]	4	3.5	39 (UV), 58 (VIS), 104 (NIR) mas	2.5
Read noise [e-]	UV: 0	UV: 0	UV: 0.008	UV: N/A
	VIS: 0	VIS: 0	VIS: 0.008	VIS: 0.008
	NIR: 2.5	NIR: 2.5	NIR: 0.32	NIR: 0.32
Dark noise [e-/s]	UV: 3e-5	UV: 3e-5	UV: 3e-5	UV: N/A
	VIS: 3e-5	VIS: 3e-5	VIS: 3e-5	VIS: 3e-5
	NIR: 0.002	NIR: 0.002	NIR: 0.005	NIR: 0.005
T_Coronagraph_ (average)	0.27	0.46	0.7	0.55
T_Opt_ (average)	UV: 0.13	UV: 0.13	UV: 0.38	UV: N/A
	VIS: 0.21	VIS: 0.21	VIS: 0.27	VIS: 0.15
	NIR: 0.3	NIR: 0.3	NIR: 0.36	NIR: 0.15

UV = ultraviolet. VIS = visible. NIR = near-infrared.

**Table 2.** Fixed Parameters Assumed in This Work and, Where Available, Their Source

Fixed parameter	Assumed value	Reference
**Reactor**		
Initial CO_2_	1 mmol/L	Minic and Thongbam (2011)
Initial H_2_	10 mmol/L	Konn *et al.* (2015)
Initial CHNOPS	10 mmol/L	See Section 2.1
Initial CH_4_	2 mmol/L	Konn *et al.* (2015)
Temperature	300 K	See Section 2.1
Pressure	250 × 10^5^ Pa	See Section 2.1
Volume	1 L	See Section 2.1
**Organisms**		
Maintenance power	5.32 × 10^−14^ J/s	Tijhuis *et al.* (1993)
Max metabolic rate	1.06 × 10^−18^ mol/L/s	Higgins and Cockell (2020)
Rate constant k	2.88 × 10^−5^ /s	Higgins and Cockell (2020)
Life span	10^6^ s	See Section 2.1
Starting population	500	See Section 3.1
**Simulation**		
Time	10^8^ s	See Section 2.1
Time step	1000 s	See Section 2.1
**Atmosphere**		Krissansen-Totton *et al.* (2018a)
CH_4_	0.1%	
N_2_	94%	
CO_2_	5%	
H_2_O	1%	
CO	10 ppm	
**Ocean**		
Surface/Floor area	3.62 × 10^7^ km^2^	Eakins and Sharman (2010)
**PSG**		
Exposure time	100 h	See Section 2.2.2

**Table 3.** Parameter Spaces Explored in This Work

Varying parameter	Assumed range
**Reactor**	
CO_2_ inflow	10^−12^ to 10^−6^ mol/L/s
H_2_ inflow	10^−12^ to 10^−6^ mol/L/s
CHNOPS inflow	10^−12^ to 10^−6^ mol/L/s
**Atmosphere**	
CH_4_ release	20%, 100%
CH_4_	0.05%, 0.25%, 1%
**Ocean**	
Vent coverage (fraction)	10^−10^ to 1
**PSG**	
Distance	5, 10, 15, and 20 pc

### 2.1. Simulation parameters

NutMEG (Nutrients, Maintenance, Energy and Growth) is a Python package for predicting the behavior of microbial organisms in astrobiology. It does so by assessing the availability of energy and nutrients and whether they meet the price for survival and growth in a given environment. It has been used previously to explore the effects of changing energy and nutrient availability for methanogens (Higgins and Cockell, 2020). A NutMEG simulation needs several input parameters to accurately model the growth of methanogens. This includes the temperature, pressure, initial concentrations of reagents (including CHNOPS elements), inflow of nutrients and substrates, metabolic rates, and energy requirements, among others. For these, hydrothermal vent fields on Earth and the characteristics of known methanogens were taken as a reference point.

Initial molecular hydrogen and methane concentrations were taken from the work of Konn *et al.* (2015) and set to be 10 and 2 mmol/L, respectively. While these are the values for abiotic molecular hydrogen and methane concentrations in hot-temperature hydrothermal vent fields, the underlying serpentinization processes leading to these also occur in lower-temperature fields such as Lost City (Lang and Brazelton, 2020). At Lost City, Kelley *et al.* (2005) describe methane concentrations between 1 and 2 mmol/kg and hydrogen concentrations up to 15 mmol/kg, while Proskurowski *et al.* (2008) report methane concentrations of 1–2 mmol/kg and molecular hydrogen concentrations up to 14.4 mmol/kg. As all of these are consistent with the values given in Konn *et al.* (2015), it was decided to proceed with these. Carbon dioxide initial concentration was set to 1 mmol/L according to Minic and Thongbam (2011).

Inflow rates of molecular hydrogen and carbon dioxide were estimated using literature values of methane production by methanogens at the Lost City hydrothermal vent field from Bradley and Summons (2010). They estimate a “low” rate of 1.2 × 10^−11^ mol/L/s and a maximum of 1.83 × 10^−5^ mol/L/s, converted to units used here. Then, a range of molecular hydrogen and carbon dioxide inflow values was tested for their corresponding methane production rates and compared to these literature values. We chose to use a logarithmically spaced parameter space ranging from 10^−12^ to 10^−6^ mol/L/s for both molecular hydrogen and carbon dioxide. This range allows for a wider variety of methanogen production rates, reaching approximately the maximum expected in Lost City and a minimum well below its “low” value. This then allows for much lower production rates than found at Lost City. The range here thus encompasses the production rates found on Earth and also includes much less favorable conditions for methanogens.

To facilitate microbial growth simulations, NutMEG also monitors the availability and uptake of carbon, nitrogen, oxygen, phosphorus, and sulfur (CHNOPS) bearing chemical species. These are the main chemical elements required to build and maintain biomass. For this work, we assumed that the nutrient-rich hydrothermal vent environments (Dick, 2019) were not limited in such elements, instead exploring the energy-limited case dictated by the inflow of molecular hydrogen and carbon dioxide. To avoid the effects of nutrient limitation on our methanogen biosphere, we include surplus CHNOPS-bearing species, including ammonia, dihydrogen phosphate, sulfate, and carbon monoxide, and instruct NutMEG to omit them from chemical analyses. Any growth limitation caused by the absence of CHNOPS elements would only increase the hydrothermal vent area needed to produce any given flux of methane.

The temperature was set to 300 K and the pressure to 250 × 10^5^ Pa, corresponding to a moderately warm deep-sea environment. These values of temperature and pressure were chosen because predictions of microbial behavior are most robust in such conditions (Higgins and Cockell, 2020). To explore a range of possible vent temperatures, we repeated our simulations at 325 and 350 K and obtained similar results for methane production rates. The reactor was given a size of 1 L in order to simplify later calculations.

NutMEG also offers flexibility in the choice of several organismic parameters. For this work, we use their default or calculated values for methanogens where available (Higgins and Cockell, 2020). We use the Tijhuis *et al.* (1993) estimate of the anaerobic maintenance power, a measure of how much energy is required per unit time for a cell to survive. These organismic parameters are important for biomass estimates, but because our goal is the total biogenic methane production, we need only a methanogen community in an approximate steady state with their environment such that the key influencer on methane production is the rate of inflow of molecular hydrogen and carbon dioxide. The life span of a typical cell was arbitrarily chosen at 10^6 seconds. This is because neither a life span nor death rate for methanogens is currently known. However, it can be shown that methane production rates can remain constant on long timescales in a steady state provided the total methanogen biomass is able to adjust to accommodate any death rate (Higgins, 2022).

Methanogen growth simulations were run for 10^8^ s, with a time step of 1000 s. This afforded the best balance of computational efficiency and precision as, to explore the parameter space, 441 simulations were run. When growth simulations entered limit cycles, the methane production rate was averaged over several cycles to obtain an approximate constant production rate.

As the carbon dioxide and molecular hydrogen availability initially increases, the methanogens are able to grow limited only by their processing speed of the substrates. However, this growth can quickly outpace the substrate inflow, leading to the active population size decreasing while substrate concentrations are too low to sustain the population. Once carbon dioxide and molecular hydrogen become available again, the cycle restarts. While this extreme cyclic model is likely unrealistic, the fact that we average over the methane production over time renders this only a minor problem. We simply demonstrate that one can use a microbial model to determine approximate methane production rates. Future analyses may be able to extend this beyond first order.

### 2.2. Atmospheric composition and spectral generation

#### 2.2.1. The atmosphere

To quantify the effect of this methane on atmospheric composition, a few parameters must be set. The first one, rate of production, has already been determined above. The composition of the atmosphere in question is also important, as it determines factors such as atmospheric lifetime. For this report, an Archean-Earth-like atmosphere was assumed similar to that of Krissansen-Totton *et al.* (2018a), with 94% molecular nitrogen, 5% carbon dioxide, 1% water, and 10 ppm carbon monoxide. Methane concentrations were varied and are described in more detail below. These values are approximate; care was taken to ensure percentages added up to 100%. We assumed a surface pressure of 1 atm, noting that literature exists arguing that lower surface pressures with higher carbon dioxide concentrations may have been present on Earth during the Archean (Lehmer *et al.,* 2020; Payne *et al.,* 2020).

The atmospheric lifetime of methane depends on the presence of oxygen in the atmosphere—in modern Earth's atmosphere, due to reaction with oxygen, methane has a lifetime of only about 10 years (Krissansen-Totton *et al.,* 2018b). In a reducing atmosphere, like the one described here, methane would be removed from the atmosphere within about 30 thousand years (Catling and Kasting, 2017, 215).

A key factor is the global rate of biological methane production. This depends on two factors: the methane production rate per liter of hydrothermal vent fluid, and the total amount of hydrothermal vent fluid. The former was computed for a range of inflow values as introduced in the previous section. The latter was determined as a fractional coverage of ocean floor. Again using Earth as a reference point, Eakins and Sharman (2010) list the total ocean surface area as 3.62 × 10^7^ km^2^—here it was assumed that ocean surface area and seabed surface area were approximately the same. Taking an average vent height of 10 m (hydrothermal vents vary in size, with some of them reaching tens of meters [Kelley *et al.,* 2001; Clague *et al.,* 2020]) makes computation of total vent volume (in liters) easily possible for a range of fractional vent ocean floor coverages. However, not all the methane produced in the deep ocean actually makes it to the atmosphere—a significant fraction is oxidized to carbon dioxide by methanotrophs (Buan, 2018). Thauer *et al.* (2008) estimated that of 1 gigaton (Gt) methane produced by methanogens, plus another 1 Gt methane released from melting methane hydrates, about 1.6 Gt is oxidized by various microorganisms, and about 0.4 Gt escapes into the atmosphere. This, however, is based on a modern Earth where aerobic oxidization contributes strongly to the removal of methane. Krüger *et al.* (2005) proposed that likely more than 80% of oceanic methane is oxidized in anoxic sediment zones. Thus, we assume here that only 20% of the methane flux produced actually makes it to the upper atmosphere.

Considering the methane production rate per time per liter as well as the total estimated volume of bioreactors in hydrothermal vent fields allows computation of a total global methane production rate from these fields. Considering the fractional outgassing of methane into the atmosphere from the deep ocean, one can then calculate the rate at which methane produced by methanogens enters the atmosphere. As we explicitly wanted to determine at what point methane concentrations would be likely biological in nature, the total methane flux considered here is taken to be the flux caused by microbial methanogenesis in the hydrothermal vent fields, plus a flat abiotic production rate, taken to encompass all non-biological sources of methane. This abiotic rate was set to 10 Tmol/year, which Krissansen-Totton *et al.* (2018b) deem to be the threshold for “likely biological.” They also demonstrate a method of linking methane flux to atmospheric methane mixing ratio. By considering the amount of H atoms in the atmosphere to be in equilibrium, they determine a linear relationship between flux and mixing ratio of approximately

$$f_{CH}=1∕3F_{CH}\left(Tmol∕year\right)∕6680\left(Tmol∕year\right)$$

where *f*_CH_ is the mixing ratio and *F*_CH_ is the global methane flux in units of teramole per year (the sum of abiotic and biotic sources). Additionally, Krissansen-Totton *et al.* (2018b) note that their original diffusion limited model likely overestimates the methane mixing ratio at a given methane flux by a factor of 2–3, since it does not account for other methane sinks. Thus, it was decided here to include an extra factor of a third in the above equation to account for this. Using this relationship, we can now directly compute atmospheric methane concentrations for a range of methane production rates at hydrothermal vent fields, and a range of fractional hydrothermal vent ocean coverage. This was done both for a “best” case, where all the methane produced gathers in the atmosphere, and a “realistic” case, where only 20% does, owing to mechanisms described above.

In the best case, a 10 Tmol/year flux, posing as a threshold between biogenic and abiotic, corresponds to a 0.05% methane concentration in the atmosphere. A 50 Tmol/year flux (“very likely biogenic”) corresponds to a 0.25% concentration. Hence, we took 0.05% methane to be the baseline (the “maximum” concentration possible that does not necessarily imply a likely biogenic origin). We compared this to concentrations of 0.25% (likely biogenic) and 1%, where 1% corresponds to a 200 Tmol/year flux, thus representing an even more probable biogenic source. These differ from the 0.1% and 1% thresholds suggested by Krissansen-Totton *et al.* (2018b), due to our inclusion of the 1/3 factor, as well as rounding errors.

#### 2.2.2. Generating spectra

Spectra were generated, as mentioned above, using NASA's Planetary Spectrum Generator^1^ (PSG). It can synthesize spectral observations from atmospheres and surfaces for a range of wavelengths (0.1 m to 100 mm) and a wide variety of current and future observing instruments (such as LUVOIR [Bolcar *et al.,* 2017] and HabEx [Mennesson *et al.,* 2016]). This is done using a combination of radiative transfer models, spectroscopic databases, and planetary databases; see the work of Villanueva *et al.* (2018) for full details. This tool also includes a simple noise calculator that takes into account both instrument configurations and detector capabilities.

As this study was based on an Archean-Earth-like exoplanet, using the modern Earth template as a starting point for the exoplanet's mass, radius, orbital parameters, and so on significantly simplified and streamlined the process (Seidelmann, 2006; Archinal *et al.,* 2018; Villanueva *et al.,* 2018; JPL, 2020; Park *et al.*, 2021). The parameters we explicitly adapted from the template included atmospheric temperatures and mixing ratios to more closely adhere to our Archean-Earth-like model. The values we altered from the template are discussed below.

The exoplanet was placed at a distance of 5, 10, 15, and 20 parsec (pc) from the observer (TRAPPIST-1 lies at about 12 pc [Gillon *et al.,* 2017]), and observed at the time when, viewed from Earth, it is farthest from its host, a Sun-like (G-type) star. This configuration is optimal when observing with a direct imaging mission such as LUVOIR or HabEx. We explored different distance ranges because distance has a large impact on the signal-to-noise ratio that can be obtained for a given spectrum.

For observation, different telescopes were used in order to allow for comparison between them: LUVOIR (LUVOIR Team, 2019), both its proposed A and B variants, observing in the UV, optical, and IR, as well as HCG (Mennesson *et al.,* 2016), both with and without the SS applied. The SS blocks light from the host star, allowing for better contrast limits. It thus permits direct imaging of faint habitable zone exoplanets. Parameters used by PSG for the generation of spectra using these instruments can be found in Table 1.

The LUVOIR (LUVOIR Team, 2019) and HabEx (Gaudi *et al.,* 2020) final mission reports detail the specifications of the instruments each mission is equipped with. A summary of the instruments and their most relevant parameters can be found in Table 1.

The exposure time was set to 100 h (Table 2), and thus signal to noise (S/N) calculated by comparing the signal achieved with the new methane concentration to the baseline signal computed with 0.1% atmospheric methane according to the formula

$$\frac{S}{N}=\sqrt{\sum_{i}{\left(\frac{S_{i}}{N_{i}}\right)}^{2}}$$

Where *S_i_* is the signal at wavelength *i* (the difference between the signal at the current methane concentration and the signal at baseline methane concentrations), *N_i_* is the noise at wavelength *i,* and the sum is performed over wavelength. We consider 5 sigma as our detection bar. Then, using this S/N, the estimated time for a 5-sigma detection (in hours) of the explored methane level compared to the baseline calculated as follows:

$$t_{5\sigma }\left(hrs\right)=100\left(hrs\right)\times {\left(\frac{5}{S∕N}\right)}^{2}$$

Surface pressure was assumed to remain constant at 1 atm. Individual gas abundances were assumed to be homogeneous across the entire atmosphere for simplicity, and as such no vertical profiles were used for gas mixing ratios or atmospheric temperature. A flat temperature of 210 K was set for the entire atmosphere (Krissansen-Totton *et al.,* 2018a) and aerosols ignored. This is a simple approach which warrants further consideration and improvement, but it was taken in order to keep the toy model simple. The implications of this are outlined in the discussion.

We computed the corresponding concentrations of the other gases in the atmosphere for a range of methane mixing ratios (Table 3), assuming abundance ratios of other gases to each other remained constant. We explored three different mixing ratios: 0.05% (abiotic/biogenic threshold), 0.25% (likely biogenic), and 1% (very likely biogenic). One major caveat is that haze will start to be produced at ∼0.5% methane concentrations for the ∼5% carbon dioxide assumed here—this will very much affect detectability, and therefore consideration of haze would lead to a more realistic model. This has been left to future work.

## 3. Results

### 3.1. Methanogen simulations

**FIG. 2.**
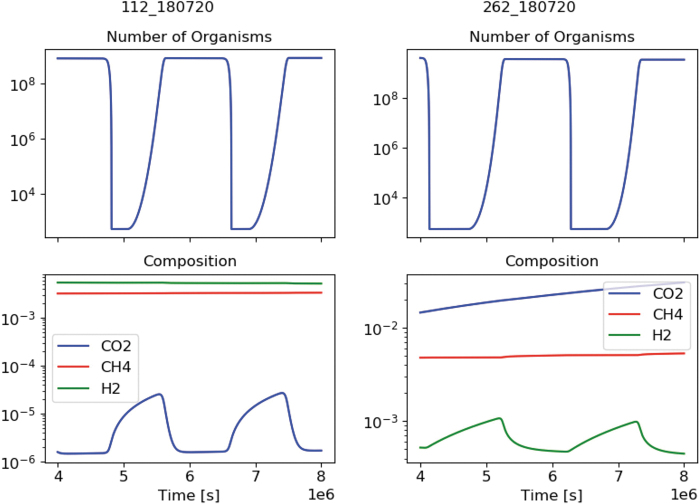
Part of the methanogen simulations, for a CO_2_-limited case (left) and a H_2_-limited case (right). The title of each graph shows the Simulation-ID as stored by the database.

Two examples of the 441 growth simulations performed are presented in Fig. 2. Only part of the total simulation duration is displayed to make the graphs clearer. The top panel displays the total number of methanogens that are currently alive, including those in a resting state, hitherto referred to as stasis (Rittershaus *et al.*, 2013). The bottom panel shows the fractional composition of the molecules which play a part in energy generation (excluding water).

In Fig. 2, the left panel (Sim 112) shows a carbon dioxide–limited case, and the right (Sim 262) a molecular hydrogen–limited case. Starting at a low population of methanogens, it can be seen that once carbon dioxide and molecular hydrogen both reach a high enough concentration, the methanogen community begins to grow—this is observed as an increase in the number of organisms. This new population has a higher energy demand, and therefore concentrations of limiting substrates drop again. If not enough energy becomes available within their life span (as is the case here), once that time has elapsed the active population decreases until a new cycle is initiated.

**FIG. 3.**
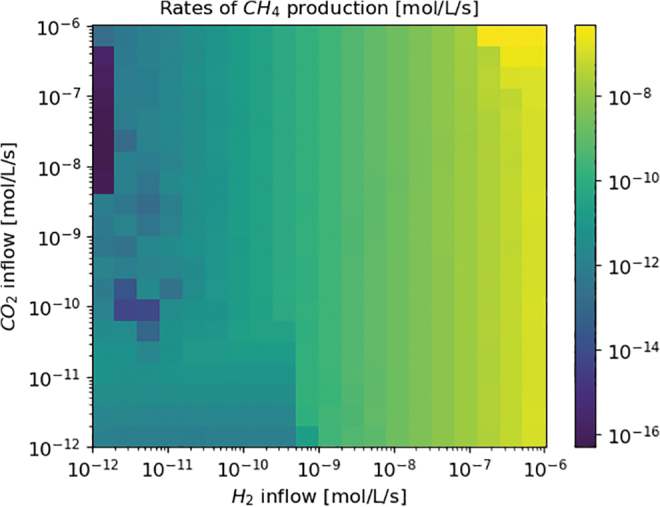
Methane production rates for varying CO_2_ and H_2_ inflow. Data was computed using the NutMEG package.

A grid of methane output rates as a function of carbon dioxide and molecular hydrogen inflow is displayed in Fig. 3. The total methane output is computed over several limit cycles, and the averaged production rate per second displayed here. It is clear that molecular hydrogen inflow has a much greater influence on methane production than carbon dioxide. This is explained by the fact that methanogenesis requires 4 moles of molecular hydrogen per mole of methane produced, but only 1 mole of carbon dioxide.

Figure 3 comprises 441 individual simulations comprising 21 different values of carbon dioxide and molecular hydrogen inflow. This number was established by the practical number of simulations (each having 10^5^ steps) that were possible using the computational resources available.

The minimum rate of production achieved over the last 10^7^ s was 4.96 × 10^−17^ mol/L/s, and the maximum 9.97 × 10^−7^ mol/L/s. The maximum compares well to the 1.83 × 10^−6^ mol/L/s observed at the Lost City system (Bradley and Summons, 2010), while the minimum is several orders of magnitude lower than the Lost City low value of 1.2 × 10^−11^ mol/L/s. This is caused by the deliberate inclusion of extreme limitation of carbon dioxide and molecular hydrogen inflow in our parameter space as it is also important to consider worst-case or sparsely populated scenarios, and whether even the related low rates of methane production could lead to a potentially detectable biosignature on an exoplanet in a later step.

Given the important role of temperature in the thermodynamics of methanogenesis, we carried out investigations as to how it could change the methane production rate. The simulations presented here were repeated at 325 and 350 K. Some variation was seen at the lowest inputs of carbon dioxide and molecular hydrogen, but above a molecular hydrogen inflow of 10^−9^ mol/L/s results were identical to those in Fig. 3. Above this value, production of methane was limited by the availability and influx of reagents in the vent, rather than the thermodynamics of the metabolism or energetic costs of survival.

### 3.2. The atmosphere

**FIG. 4.**
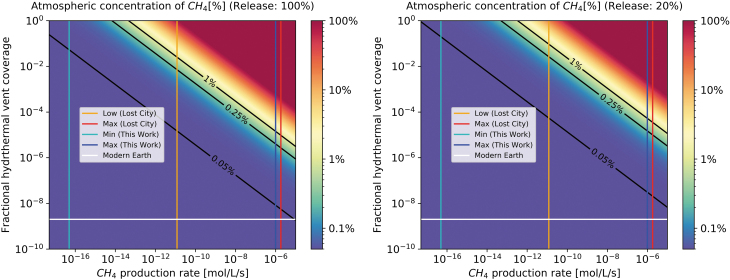
Atmospheric methane concentration for a range of different methane production rates and fractional hydrothermal vent coverages. The left graph assumes complete outgassing, while in the right, only 20% of methane produced actually accumulates in the atmosphere. The black diagonal lines indicate the baseline methane concentration of 0.05% as well as a threshold “likely biogenic” methane concentration of 0.25% (calculated using an adaptation of the formula described in Krissansen-Totton *et al.* [2018b]), and a 1% atmospheric methane threshold. Orange and red lines show the low and maximum methane production rates at Lost City, respectively, while cyan and blue indicate the minima and maxima (respectively) as computed in this work. The white line shows a rough estimate of the fractional hydrothermal vent coverage on modern Earth.

Figure 4 shows methane concentrations in the atmosphere for a range of hydrothermal vent coverage fractions and methane production rates achieved by the methanogens, plus the baseline abiotic flux of 10 Tmol/year. The left panel here assumes an unrealistic best-case scenario of complete outgassing and accumulation, while the right displays a more conservative case, in which only 20% of the methane flux makes it to the atmosphere (Krüger *et al.,* 2005). It is clear that an interplay of high hydrothermal vent coverage and moderate to high methane production rates is necessary to reach the 0.25% “likely biogenic” methane threshold proposed by Krissansen-Totton *et al.* (2018b), indicated here as a black line. Even at maximum production rates determined in this work, a coverage fraction of approximately 4 × 10^−4^ % is necessary for complete outgassing, and an even higher fraction of 1.5 × 10^−3^ % for the more conservative scenario. On the other hand, even a 100% hydrothermal vent coverage would not be sufficient to break the threshold at the lowest methane production rates computed in this work—at complete coverage, approximate production rates of 7 × 10^−15^ mol/L/s (best case) and 5 × 10^−14^ mol/L/s (conservative case) are required to reach the threshold.

#### 3.2.1. Comparison to modern Earth

It can be useful at this point to draw a comparison of this work to modern Earth conditions, in order to better illustrate the consequences of some of the findings presented here.

Baker *et al.* (2016) surveyed 1470 km of ridge systems, finding 184 hydrothermal vents, or one vent every 2 to 20 km. Assuming this sample is representative of ocean ridges on Earth in general, for 6600 km of total ridge system, this translates to a little more than 8000 vents on this planet. With an assumed area of 10 by 10 m per vent, this implies only around 2.3 × 10^−7^ % of the ocean floor is covered in hydrothermal vents—marked as a white line in Fig. 4. This is an approximation, but it shows that even at maximum methane production rates found at Lost City, not enough methane would be produced by deep sea methanogenesis in hydrothermal vents alone to provide a biosignature. However, this vent coverage is based on vent production on modern Earth. An exoplanet more similar to an Archean Earth might potentially be much more geologically active and thus host many more hydrothermal vents, as Archean Earth likely did.

Considering production rates from the Lost City hydrothermal vent field (1.2 × 10^−11^ low, 1.83 × 10^−6^ maximum [Bradley and Summons, 2010], marked as orange and red lines in Fig. 4, respectively), it can be noted that hydrothermal vent coverage fractions of 0.7 % and 0.00027% are necessary, respectively, to produce atmospheric methane concentrations of 1% (in the aforementioned best-case scenario of complete outgassing). Comparing this to modern Earth's here estimated 0.00000023% coverage, it is clear that a potential exoplanet with vents operating similarly to the Lost City field would need to be very geologically active in order to provide sufficient methane flux.

The global carbon project (Saunois *et al.,* 2020) estimates the current methane production rate on Earth to be about 600 Mtonnes/year, which corresponds to 37.4 Tmol/year, or 1.19 × 10^6^ mol/s. This is close to the 50 Tmol/year determined by Krissansen-Totton *et al.* (2018b) to lead to 1% methane in the atmosphere. However, methane concentrations in the current atmosphere are about 1.85 ppm (Nisbet *et al.,* 2019)—Krissansen-Totton *et al.* (2018b) note that their formula likely overestimates the methane concentration in the atmosphere. This much lower value is caused by two factors: Firstly, as mentioned, in oxic atmospheres, like that of modern Earth, methane has a short lifetime of about 9 years, meaning it has less time to accumulate. Secondly, there are other methane sinks present on Earth, such as soils. These account for approximately 30 Mtonnes/year, or 1.87 Tmol/year (Saunois *et al.,* 2020). This is small compared to the atmospheric reaction sink.

### 3.3. Spectra

#### 3.3.1. LUVOIR and HabEx concepts

Now that it has been determined which different environmental factors allow high concentrations of biogenic methane to be accumulated in the atmosphere, we can consider whether or not these concentrations of methane are feasibly detectable.

**FIG. 5.**
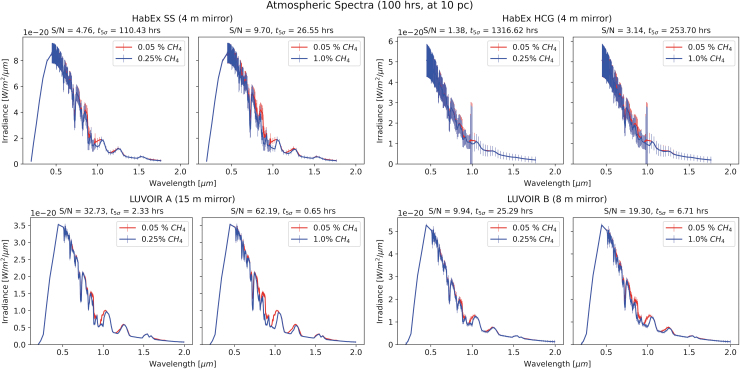
Transmission spectra of the synthetic exoplanet for a 100 h exposure at 10 pc for different CH_4_ concentrations. Top panels show spectra acquired with HabEx (with SS on the left, without on the right), bottom with LUVOIR (A on the left, B on the right). Signal-to-noise ratios are shown above each spectrum, as well as times to a 5-sigma detection of the signal. We see that, for HabEx, the inclusion of the SS vastly improves the S/N. In a similar vein, the larger mirror of LUVOIR A compared to LUVOIR B also leads to better S/N. Here, the larger 1% methane biosignature allows for detection within less than 30 h for all instruments but HabEx without the SS. The two LUVOIR variants manage detection times <7 h, owing to their larger mirrors when compared to HabEx. The 0.25% signature is much more difficult to detect for all instruments, leading to necessary exposure times of over 1000 h for HabEx without the SS and just over 100 h with the SS. Both LUVOIR models are capable of detecting this signature in less than 30 h, with LUVOIR A being about an order of magnitude faster, allowing detection in just a few hours.

**FIG. 6.**
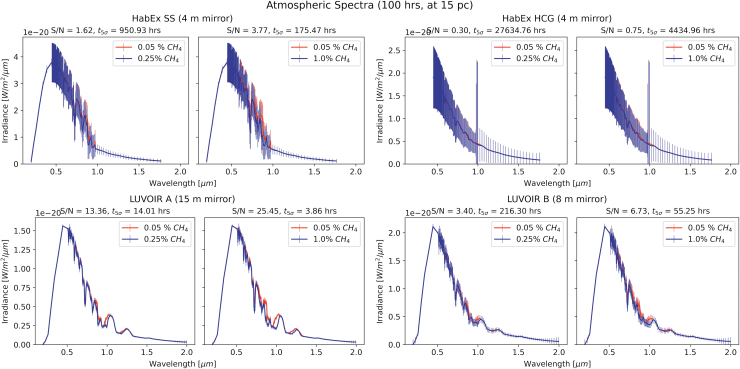
As above, at a distance of 15 pc. We note that detecting the larger 1% methane biosignature is feasible within less than 200 h of observation with all instruments except the no-SS HabEx. The more subtle 0.25% methane biosignature requires the large mirror of LUVOIR A for a detection time in the double digits (in hours), potentially rendering this detection infeasible with all but the largest mirrors. This stands in contrast with the 10 pc (or even 5 pc) case, where much shorter timescales can lead to confident detection of signatures.

Figures 5 and 6 show spectra for atmospheric concentrations of methane of 0.25% and 1%, compared to the assumed baseline of 0.05%, for a 100-hour exposure at 10 and 15 pc, respectively, bracketing the distance to the TRAPPIST system. The top row displays the spectra as taken by HabEx (with SS on the left, without on the right) and the bottom by LUVOIR (A on the left, B on the right). While these do not cover exactly the same wavelength range, there is significant overlap. Of course, covering a larger wavelength range potentially leads to inclusion of more biosignature features; thus, ideally, the range covered should be as large as possible.

Figures 5 and 6 show that the detection of methane using HabEx is generally more difficult than using either LUVOIR configuration. At all distance scales, HabEx SS takes at least an order of magnitude longer than LUVOIR A—HabEx without the SS between 2 and 3 orders of magnitude. This is not surprising; HabEx's mirror is 4 m in diameter, while LUVOIR A and B utilize a 15 and 8 m mirror, respectively, allowing them to collect more photons in the same time and thus reach a better S/N.

Further, these plots demonstrate the tremendous effect of distance on the S/N and thus the detectability of biosignatures. All observation times to detection worsen significantly in some cases by more than an order of magnitude, despite only a 50% increase in distance. As such, if we wish to explore faraway exoplanet atmospheres, it is particularly important to pay close attention to instrument performance.

**FIG. 7.**
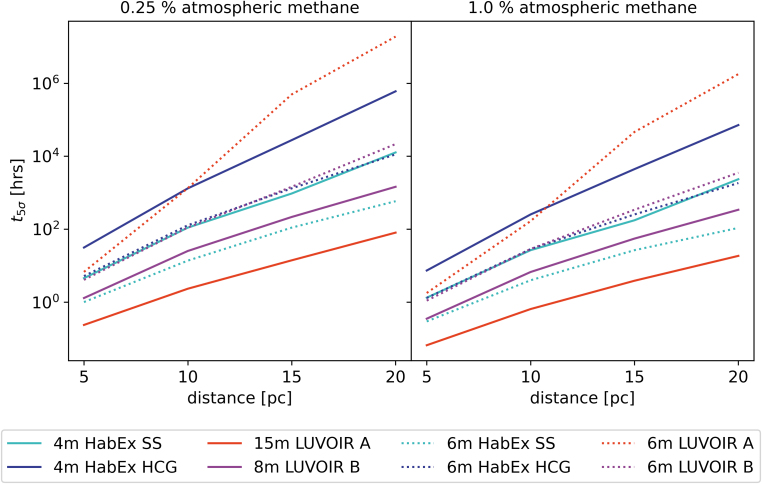
Comparing the time to a 5-sigma detection for different instruments for a range of distances. The vertical axis shows the time to a 5-sigma detection in hours on a log scale, while the horizontal axis displays distances of 5–20 pc, in 5 pc increments. Different-color lines indicate different instruments, with solid lines signifying the instruments at their “native” size and dotted lines showing the instruments rescaled to a 6 m mirror. We observe that the time to detection seems to grow roughly linearly in this log plot, implying an exponential relationship between distance and detection time. Additionally, the choice of instrument shifts this line up or down but does not seem to have a major impact on its slope (with the exception of 6 m LUVOIR A). Turning our attention to the dotted lines indicating 6 m versions, we see that the scaled HabEx with the larger mirror (cyan, dotted) performs on par with, or better than, all but the largest proposed instrument, LUVOIR A. At shorter-distance scales, it performs very similarly to an 8 m LUVOIR B. A 6 m HabEx without the SS (blue, dotted) and 6 m LUVOIR B (magenta, dotted) perform very similarly on shorter-distance scales but diverge more toward larger distances. Their performance is comparable to an unscaled 4 m HabEx with the SS (cyan, solid). Overall, it appears the inclusion of a SS leads to similar gains in performance as increasing the mirror diameter by 2 m, as indicated by 4 m HabEx SS (cyan, solid) and 6 m LUVOIR B (magenta, dotted), as well as 6 m HabEx SS (cyan, dotted) and 8 m LUVOIR B (magenta, solid) performing very similarly.

Figure 7 shows the scaling of the observation time to detection for each instrument with the distance, further underlining the effect of aperture size on an instrument's capability to detect methane. Instruments with larger mirrors generally outperform those with smaller mirrors at all distance scales. Additionally, HabEx with the SS yields consistently lower detection times than without the SS, despite an identical mirror size. This can be attributed, in part, to the SS blocking much more of the hypothetical host star light than a coronagraph alone. This leads to a less contaminated view of the planet and thus a better S/N.

#### 3.3.2. The LUVEX concept

In light of the NASEM Astronomy and Astrophysics decadal survey's (NASEM, 2021) recommendation of a hybrid between LUVOIR and HabEx, Fig. 7 also explores how each instrument would be expected to perform for the science case presented therein, were its mirror rescaled to 6 m (the size suggested by the decadal). We do note the major caveat that these are only rough, approximate predictions based on PSG and that in reality a change of mirror would very likely also come with different instrument configurations and noise profiles. However, we deemed this avenue worth exploring as a preliminary look into the potential performance of these proposed instruments.

In line with the previous plots, the SS very much improves HabEx's performance compared to the version without the SS. This, combined with the larger mirror diameter of 6 m, makes the HabEx variant the second-best choice in this scenario, losing only to the original 15 m LUVOIR A, which uses a mirror over twice as large in diameter. Even then, the scaled HabEx SS remains within an order of magnitude of 15 m LUVOIR A for all distances. It outperforms 8 m LUVOIR B by roughly a factor 2, and its own smaller version, 4 m HabEx SS, by approximately a factor 8 at distances comparable to the TRAPPIST system. Compared to the small, no-SS 4 m HabEx model, this variant manages to decrease detection time by about 2 orders of magnitude at these distance scales.

It must be noted at this point that the mirror size is not the only governing factor in instrument performance and that different technologies used in different instruments have different associated noise profiles. These noise profiles also have an important effect on the S/N achievable by the instrument.

**FIG. 8.**
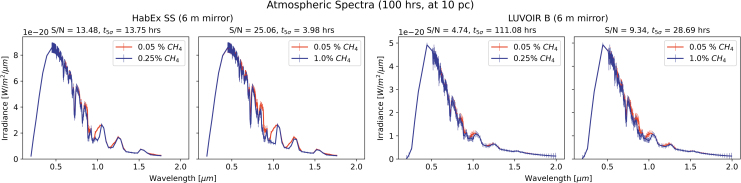
Similarly to Figs. 5 and 6, we now compare a rescaled HabEx with the SS to a rescaled LUVOIR B, at a distance of 10 pc. With the mirror size no longer a deciding factor between the instruments, we see that HabEx with the SS actually outperforms LUVOIR B of the same size. Additionally, HabEx now manages to detect the stronger 1% signature in just under 4 h, even faster than an 8 m LUVOIR B in Fig. 5. Both instruments also perform reasonably in the more difficult to detect 0.25% methane scheme, with 6 m LUVOIR B taking just over 100 h for detection and 6 m HabEx SS just over 10.

With the aforementioned in mind, it is useful to compare the spectra achieved by HabEx with the SS and a 6 m mirror, to LUVOIR B with a 6 m mirror. Figure 8 shows the spectra as generated by these rescaled instruments, for both the 0.25% and the 1% methane case. The larger HabEx SS now outperforms the downscaled LUVOIR B. However, both demonstrate good observation times to detection for the stronger signature at 10 pc and still reasonable times for the weaker 0.25% signature. Thus, the decadal recommission should permit for the pursuit of biosignatures, though we note the inclusion of a SS still decidedly improves the instrument's performance. All but the largest instruments struggle to detect the more subtle biosignature at the largest distances, but the second-best performance achieved by a comparatively small 6 m HabEx SS over all distances lends credence to the efficacy of this concept.

## 4. Discussion

In this study, we attempted to bridge the gap between microbiological simulations and the analysis of atmospheric spectra using a ground-up model to estimate methane production and propagation from a biological source—methanogenic activity. A combination of large hydrothermal vent coverage (in comparison to modern Earth) and frequent supply of carbon dioxide and molecular hydrogen to methanogens is necessary to achieve “likely biogenic” atmospheric methane levels of 0.25%. However, to confidently detect methane at distances comparable to the TRAPPIST system (10–15 pc), careful instrument planning and design is required, if observation time to a credible detection is to be kept to a minimum. As this result is derived from a combination of models, there are a number of caveats associated with the analysis. These are addressed here, in order of calculation.

### 4.1. Methanogen simulations

The temperature was assumed to be 300 K. This was selected as a compromise between the hot white smoker effluent itself (at 40–90°C) and the cold seawater in the deep ocean (2°C) (Ridgway, 1969), as effluent and seawater likely mix in the area surrounding a hydrothermal vent, forming the habitable area. In reality there is a temperature gradient, rather than a constant field of moderate temperature. Our temperature choice of 300 K will yield the most accurate thermodynamic calculations and most reliable estimate of organism behavior, however. As seen in the work of Higgins and Cockell (2020), temperature affects biomass and biosignature production exponentially in closed systems, but our results show that with a significant enough inflow of molecular hydrogen, the temperature choice does not affect methane production because the total biomass will adjust to make the most of any available energy. We note however that in a more realistic system the inflow of carbon dioxide and molecular hydrogen will be dependent on the temperature of the vent and composition of the rock, meaning, for example, as temperature increases the environment is more likely to be represented by the right and top of Fig. 3.

The transfer of methane to the outside of each 1 L reactor was greatly simplified. As NutMEG only allows for a constant in- or outflow of molecules, and the production rate of methane changes significantly over time (particularly at the beginning), no realistic methane outflow could be programmed into the simulation. Instead, the methane was allowed to accumulate for 10^8^ s, under the assumption that this accumulation would not markedly impact production rates as the forward-reaction becomes less and less energetically favorable. Releasing all the methane immediately after the elapsed time could be another source of issues, as the atmospheric accumulation model used assumes constant production. However, within the assumed timescales (30,000 years), the running time of the simulation (a little over 3 years) makes this issue less of a concern. In the future, ideally a variable outflow of molecules would be included in the simulation, to vary methane outgassing with methane production and avoid its accumulation.

NutMEG best handles large numbers of organisms as a community, as was done here. This means that individual members are not tracked, but rather the behavior of the group as a whole. This has a number of effects, most notably the effects of the life span on the model calculations. When the life span of a species has elapsed, the number of organisms that were created that life span ago are removed. This, together with the comparatively still large time step of 1000 s, leads to large drops in the population as all the organisms created in the initial “growth phase” are removed within a few steps, which is not reflective of the typical death phase of microbial growth. Individual tracking, as well as smaller steps, would allow for less extreme reductions in population, but at the cost of computational efficiency.

Additionally, the initial population of methanogens (here 500) is unaffected by the life span, which leads to some important computational implications, as the whole population of methanogens can never completely “die out.” This is unrealistic, as extreme nutrient or substrate limitation could lead to a total extinction of methanogens in a given simulation, while having a persistent starting population allows for eventual recovery, circumstances permitting, such that we could compute the time-averaged methane production. This caveat and the one above regarding life span have been resolved in a newer version of NutMEG (version 1.0.0 +) which incorporates a death rate instead of a life span (Higgins, 2022). We used version 0.9.0 in this study to best corroborate with the methanogenesis results from Higgins and Cockell (2020). We expect these caveats to have negligible effects on the time-averaged methane production rates.

Simulations were loosely based on conditions found at the Lost City hydrothermal vent field. There are, however, much hotter hydrothermal vent types (black smokers), which are home to thermophilic or hyperthermophilic methanogens, which may exhibit different, potentially higher, production rates.

### 4.2. Accumulation in the atmosphere

Although we consider end-member atmospheric accumulation of the methane generated of 100% and 20% as maximum and more conservative values, respectively, we acknowledge that this range could be refined if we had more knowledge of the planetary environment. For instance, one could include other methane sinks such as methylotrophs in the ocean above the hydrothermal vents. Ultimately, the transport of biologically useful and chemically reactive species through exoplanet oceans, soils, and/or atmospheres will depend on the unique ecosystem of that body. Hence, this end-member approach should provide a plausible range for a variety of possible exoplanet ecosystems.

As has been mentioned before, a major simplification made here was the assumption of mixing ratios that are constant with altitude. In reality, different molecules have different mixing ratios at different heights. A thorough photochemical model would allow for more accurate computation of mixing ratios as a function of altitude, which would have significant effects on the spectra generated here. For example, methane concentrations fall dramatically at high altitudes, meaning higher overall methane productions would be required to show a comparably strong methane presence in the spectrum. However, such photochemical analysis is beyond the scope of this work. In addition, aerosols and clouds were neglected for simplicity, and a flat temperature of 210 K for the entire atmosphere set (Krissansen-Totton *et al.,* 2018a).

Specifically, hydrocarbon haze has been neglected in this work. At the atmospheric compositions considered here, the methane–carbon dioxide ratio crosses the threshold of hydrocarbon haze production (Pavlov *et al.,* 2001). According to Arney *et al.* (2016), this haze has a big impact on planetary spectra and thus, ideally, needs to be considered when computing atmospheric profiles and mixing ratios.

### 4.3. Spectra generation

When considering which instrument with which to perform observations, a number of factors have to be taken into account (Pidhorodetska *et al.,* 2020). Below, we discuss a few of them in some more detail.

**Wavelength range**: Neither HabEx nor LUVOIR cover the range of 2–12 μm, within which two important methane features can be found.

**Aperture size and noise:** As has been discussed in Section 3, a larger aperture implies more photons collected, leading to better S/N. The signal scales with *n,* while the noise scales with $\sqrt{n}$, meaning the S/N is proportional to $\sqrt{n}$, where *n* is the number of collected photons, which goes as the square of the radius of the aperture. However, a larger mirror also quickly increases the cost of the instrument—as such, a balance must be struck.

**Resolution:** As can be seen in Figs. 5 and 6, the instruments in question here have different resolving powers in different regimes. At very low resolutions, narrow features might be obscured that would otherwise be visible at higher resolutions.

Specific attention was given to methane as a biosignature in this study. It was selected as the primary output of a simple, well-understood metabolism to demonstrate that microbiological simulations can be extrapolated to the planetary scale and linked to possible observations. There are a huge number of other biosignature candidates to consider (*e.g.,* Seager *et al.,* 2016; Schwieterman *et al.,* 2018), many of which could have stronger spectral presence requiring lower atmospheric levels for confident detection. Our approach sets the precedent that this can likely be repeated for other organisms, environments, and the relevant biosignatures.

Finally, explored here is the detectability of methane—though methane individually does not necessarily imply an active biosphere. Detection of carbon dioxide and other biosignatures alongside likely biogenic methane levels is important to more confidently suggest the existence of life on a planet.

## 5. Further Notes

To enable the analysis present in this work, a number of major simplifying assumptions had to be made that very likely impact the accuracy of the model used here. The goal of this work was to attempt a bottom-up investigation of the detectability of methane produced at hydrothermal vent sites as a biosignature on an Archean-Earth-like exoplanet. Thus, these simplifications were tolerated for the purpose of providing an analysis of the process end-to-end, albeit with several important factors which require more detailed consideration. The points mentioned in this section provide a non-exhaustive list of potential shortcomings of this work that call for further examination in future projects.

As has been mentioned, the formation and impact of haze on the detectability of methane has been ignored. This is a caveat of this work, as at some of the atmospheric compositions explored here, haze formation would likely occur. Haze, on an Archean-Earth-like planet, would likely provide a cooling effect on the surface. This has been assumed to cause the planet to freeze, while Arney *et al.* (2016) suggested that haze may actually be beneficial to sustaining life on the planet, as the cooling effect is self-limiting, and the haze blocks out much potentially hazardous UV flux. Further, the methane fluxes required to sustain haze formation in Archean-Earth-like conditions are consistent with estimated biological production rates (Arney *et al.,* 2018). This haze is potentially observable with instruments such as the James Webb Space Telescope, providing a possible biosignature for planets similar to early Earth (Arney *et al.,* 2017).

It is also important to consider the wider effects of these methane abundances, such as the climatic effects of high atmospheric methane concentration, ocean methane cycling, and atmosphere-ecosystem interactions. Kharecha *et al.* (2005) provided an overview of an Archean-Earth atmosphere-ecosystem model, including the production and destruction of methane. Further early-Earth climate models can be found in the works of Olson *et al.* (2016) and Ozaki *et al.* (2018), with the latter taking early photosynthetic life into account. Reinhard *et al.* (2020) presented a methane cycle model with a range of applications, showing good agreement of simulated methane levels with empirical modern Earth values.

Lastly, and perhaps most crucially, the issues with false positives and false negatives should be emphasized. Reinhard *et al.* (2017) discussed the problems of false negatives, pointing out that early Earth itself had a methane biosignature that would have been problematic to detect for much of its history. Wogan *et al.* (2020) investigated the possibility of a large non-biological methane flux from volcanism, presenting one possible false-positive diagnostic.

## 6. Conclusion

In this work, we combined microbiological simulations with atmospheric spectral analysis, an area of research that promises to play a key role in the search for biosignatures on exoplanets. Focus was placed on the production and detectability of methane as a biosignature on an Archean-Earth-like exoplanet. Recent findings by Rucker *et al.* (2022) suggest that hydrothermal vent systems which have high potential for biological methane production are expected to be pervasive on rocky planets. Thus, a wealth of Earth-like and non-Earth-like systems can be explored using models like the one presented in this work.

It was found that, at hydrothermal vent coverages comparable to modern Earth, no amount of computed microbial methane production would be sufficient to lead to a detectable methane signature in the atmosphere. Thus, such a biosignature would have to occur on a planet much more geologically active than modern Earth, or be subsidized by other biogenic sources of methane, which were not explored here. Additionally, large-enough molecular hydrogen inflow rates in particular are required at vent sites in order to allow for significant methane production by methanogens.

With these conditions met, telescope size, the inclusion of a SS, and other instrument choices become very important. We have demonstrated that a larger mirror, provided everything else remains the same, is beneficial to the detectability of a methane biosignature. Further, distance to the exoplanet in question has a large impact on how quickly a biosignature can be confidently detected at 5 sigma, as, regardless of instrument choice, detection times rapidly increase with distance. The HabEx/LUVOIR hybrid proposed by the Astronomy and Astrophysics decadal survey is capable of finding biosignatures within reasonable time frames at distance scales comparable to our separation from the TRAPPIST-1 system, though we note the inclusion of a SS vastly improves the instrument's performance, leading to a reduction of the detection time of a factor 2 compared to the next-best instrument (8 m LUVOIR B) and a factor 8 compared to its own, smaller version (4 m HabEx SS). Only the over twice as large 15 m LUVOIR A manages to beat this performance, by approximately a factor 8.

This work made a number of assumptions about the biological production of methane and its accumulation in the atmosphere. It did not include photochemical models or other methanogenic metabolisms, for instance. The lack of rigorous photochemical modelling is one of the major simplifications made here. Mixing ratios of gases such as methane vary with height in the atmosphere. Hence, while it enables a sweeping analysis of the process of biosignature production, it is not entirely reflective of reality. Nevertheless, this work illustrates how microbial ecological modelling can be coupled to exoplanetary spectral models to arrive at first-order estimates of the detectability of exoplanet biosignatures. As such, it shows one way in which the fields of microbiology and exoplanet science must collaborate to advance our capacity to interpret spectral data obtained from exoplanets in the habitable zone.

## Abbreviations Used

CHNOPS: carbon, nitrogen, oxygen, phosphorus, and sulfur
HabEx: Habitable Exoplanet Observatory
HCG: HabEx Coronagraph
LUVEX: LUVOIR/HabEx hybrid
LUVOIR: Large Ultraviolet/Optical/Infrared Surveyor
NASEM: National Academies of Sciences, Engineering, and Medicine
NutMEG: Nutrients, Maintenance, Energy and Growth
PSG: Planetary Spectrum Generator
S/N: signal to noise
SS: starshade

## Notes

Associate Editor: Christopher McKay

## Notes

^1^

https://psg.gsfc.nasa.gov
